# Climate and Other Human Causes of Spatial Variation in Mallard Mortality and Population Dynamics

**DOI:** 10.1002/ece3.74596

**Published:** 2026-10-29

**Authors:** Madeleine G. Lohman, Thomas V. Riecke, James S. Sedinger, Perry J. Williams

**Affiliations:** ^1^ Department of Zoology and Physiology University of Wyoming Laramie Wyoming USA; ^2^ Department of Ecosystem and Conservation Sciences University of Montana Missoula Montana USA; ^3^ Department of Natural Resources and Environmental Science University of Nevada Reno Nevada USA

**Keywords:** avian, Bayesian, demography, mallard, modeling, spatial modeling, spatial statistics, waterfowl

## Abstract

Wildlife population dynamics change across time and space, but few studies use formal spatiotemporal statistical models to understand these patterns. The population distribution of mallards (*Anas platyrhyncos*) in the Prairie Pothole Region of central North America (PPR) has shifted dramatically over the past several decades as agricultural intensity and climate have changed. We examined spatiotemporal variation in survival, natural mortality, and harvest mortality rates for mallards in the PPR from 1974 to 2023 using intrinsic conditional autoregressive models in a Bayesian framework. There was substantial variation over time and space for all demographic rates, and relationships between mortality and habitat variables varied by age and sex cohort. Specifically, environmental relationships indicate that habitats with less agricultural activity and more wetlands decrease survival via increased natural mortality driven by reproductive risks. We also found a worrying trend: females exhibited declining survival probabilities since the late 1980s and early 1990s. Our analyses demonstrate major variation in survival rates of a well‐studied organism across an environmental gradient, reveal subtle but critical differences relative to inference obtained from pooled data, and provide a clear roadmap to future research that can inform spatially explicit conservation and management goals.

## Graphical abstract

We examined spatiotemporal variation in survival, natural mortality, and harvest mortality rates for mallards in the PPR from 1974 to 2023 using intrinsic conditional autoregressive models in a Bayesian framework. Environmental relationships indicate that habitats with less agricultural activity and more wetlands in breeding grounds may benefit dabbling duck populations via increased fecundity, but decrease survival via increased natural mortality driven by reproductive risks. We also found a worrying trend: females exhibited declining survival probabilities across the time series.

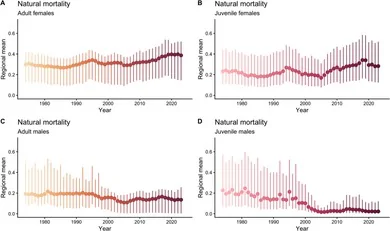

## 1 Introduction

Population dynamics and distributions vary across time and space. This variation in demography often results from spatial and temporal variation in biotic and abiotic conditions, which can complicate the conservation and management of species. Such variation, however, is essential for understanding factors that regulate population dynamics (Kareiva 1990; Lawson et al. 2015; Tuljapurkar 2013). Much research has focused on understanding how populations have evolved in response to environmental variation (Kareiva 1990; Levin 1976; Whittaker and Goodman 1979) and examining existing variation in life‐history strategies through this lens (Nichols et al. 1995; Stewart and Kantrud 1973; Wolff 1980).

Rapid habitat changes can alter species interactions, the variability of demographic rates, and life history trade‐offs, creating a complex web of interactions between environmental conditions and demography (Gamelon et al. 2017). Direct human sources of mortality, such as hunting and trapping, and their implications for demography have received substantial attention as well (e.g., Johnson et al. 1997; Nichols et al. 1995). Harvest can alter processes associated with population dynamics (Johnson et al. 1997; Nichols et al. 1995), though evidence is mixed about the impact of harvest at the range‐wide scale (Sedinger and Herzog 2012; Riecke, Lohman, et al. 2022). However, changes in land use and climate have led to unexpected responses of populations to harvest (Peeters et al. 2022; Riecke, Sedinger, et al. 2022). For instance, Peeters et al. (2022) found that harvest buffered reindeer populations against environmental change by decreasing the impacts of resource competition. These co‐occurring changes may require a more local and nuanced examination of population dynamics than was historically necessary as environmental variation increases over space and time (Sæther et al. 2006; Zhao et al. 2016).

Substantial intra‐population variance in genetics or local habitat conditions may affect the results of smaller‐scale studies examining the role of the environment in generating variation in population dynamics. Thus, when examined piecemeal, relationships between demographic rates and environmental shifts may not hold true for the broader population. Similarly, pooling data across a broad landscape may obscure smaller‐scale shifts that consistently influence demographic trends of a certain species. Despite extensive research examining how environmental variation affects population trajectories and demographic rates (e.g., Ryan et al. 1998), researchers have conducted few studies on continuous landscape‐level scales (but see Buderman et al. 2020; Janke et al. 2017; Specht and Arnold 2018; Weegman et al. 2024) or in a spatially explicit framework (though see Royle and Dubovsky 2001). Spatially explicit models directly address this problem by allowing for finer‐scale variation in demographic rates within a broader landscape‐scale context that can better account for processes that happen on specific scales and target management resources towards areas most in need of them.

Despite the increasing interest in using spatially explicit models to examine variation in demographic rates, these tools have rarely been applied (see Holmes 2005; Sauer et al. 2017), and application to demographic data is rarer still (Frederiksen et al. 2014; Royle and Dubovsky 2001; Saracco et al. 2010). When demographic data are spatially clustered but environmental covariates vary across the landscape, non‐spatial models that pool across regions may obscure or bias relationships between environment and demography. Spatially explicit models provide a framework to address this mismatch. In Saracco et al. (2010), researchers used spatially explicit estimates of residency and survival probabilities for Wood Thrushes ( *Hylocichla mustelina* ) to identify areas of high‐ and low‐quality habitats. In addition to revealing information about the basic biology of that species, these estimates can aid in making informed decisions about where habitat improvement is most necessary. Similarly, recent work by Eisaguirre et al. (2025) used spatially explicit models to examine relationships between space use and mortality for willow ptarmigan and black bears. The results of these studies (Eisaguirre et al. 2025; Saracco et al. 2010) provide critical context for informed conservation actions, yet the use of spatially explicit models for demographic inference remains limited.

The Prairie Pothole Region (PPR) is a critical breeding habitat for waterfowl and other migratory birds, supporting over half of the breeding ducks in North America (Batt et al. 1989; Janke et al. 2017). This large region of the northern Great Plains encompasses a variety of habitat types and climatic patterns (Samson and Knopf 1996). For example, drought patterns interact with other abiotic conditions, such as temperature, to create fluctuations in wetland habitat availability, which then generate fluctuations in duck survival and reproductive success (Johnson and Grier 1988; Zhao et al. 2016; Riecke, Sedinger, et al. 2022). Variation in recruitment subsequently influences survival rates as females invest more energy in fecundity (Devries et al. 2003; Singer et al. 2016). Humans have introduced further heterogeneity into the landscape through the expansion of agriculture: three‐quarters of native uplands (Samson and Knopf 1994) and half of native wetlands (Dahl 1990) have been converted to agriculture or drained (Anteau 2012; Anteau et al. 2016). In the southeastern portion of the PPR (i.e., eastern Dakotas), agriculture has long dominated the region, draining many of the abundant ephemeral pothole ponds and transforming prairie habitat (Samson and Knopf 1994; Samson and Knopf 1996). However, agricultural subsidies beginning in the mid‐1980s have facilitated wetland conservation, increasing the amount of wetland and grassland habitat via mechanisms such as the Conservation Reserve Program (Drever et al. 2007; Kantrud 1993; Reynolds et al. 2001). In contrast, the northern portion of the region (i.e., Canada) historically possessed a higher proportion of native habitat and denser mallard ( *Anas platyrhynchos* ) populations (Drever et al. 2007; Janke et al. 2017). However, agriculture in the region expanded and intensified from the early 1960s to the mid‐1980s (Bethke and Nudds 1995), and continues. Agricultural expansion refers to the increasing acreage of agriculture across the landscape, while intensification to practices such as the reduction of fallow land and the increase of use in pesticides. Subsequently, there has been a corresponding shift in mallard density from the Canadian prairies to the eastern Dakotas (Janke et al. 2017). These land‐use gradients, combined with natural heterogeneity in the landscape, create a “natural experiment” to examine how changing environmental conditions affect the dynamics of mallard populations.

Waterfowl are among the most closely monitored vertebrate taxa in North America, resulting in extensive longitudinal data on ducks in the US and Canada (USGS Bird Banding Laboratory 2025). We examined spatiotemporal variation in demographic rates for a widespread waterfowl species, the mallard, in the PPR from 1974 to 2023 to understand changes in population dynamics in response to a changing environment. Vital rates vary among age‐ and sex‐classes or cohorts (Burnham et al. 1984; Yetter et al. 2018) and with environmental conditions (Janke et al. 2017; Miller 2000). Adult and juvenile females face increased risks associated with reproduction relative to males, so their survival may vary substantially with changes to breeding habitat (Brasher et al. 2006; Riecke, Sedinger, et al. 2022). In contrast, juvenile males face limited reproductive costs during their first potential breeding attempt, so their survival may be influenced less by factors that incite adult females to invest more effort in reproduction.

While dispersal probably contributes to some variation in dabbling duck population change, previous research has shown that mallards and other species demonstrate relatively high levels of fidelity to their breeding areas (Arnold and Clark 1996; Doherty et al. 2002; Cunningham et al. 2016). Doherty et al. 2002, also working at a landscape‐level scale, reported an average philopatry rate of 0.85 for adult females, 0.7 for juvenile females, 0.82 for adult males, and 0.31 for juvenile males. So, while there is certainly movement away from breeding areas (especially by juvenile males), broad spatiotemporal trends in demographic rates and landscape‐level variation in population dynamics are likely a function of the demography of individuals hatched and breeding in a specific area. To date, evidence suggests that landscape‐level changes have strongly impacted the distribution and abundance of mallards in the PPR. Since the early 1960s, “hot‐spots” of mallard distribution have shifted, moving from the northwestern portion of the region in Canada to the southeast in the United States (Janke et al. 2017). Previous studies attributed changes in mallard distribution and population growth to altered landscapes and weather (Janke et al. 2017; Miller 2000). Intuitively, these shifts in population density likely result from changes in survival and recruitment across time and space. Thus, researchers can attribute a substantial portion of the spatiotemporal variation in demographic rates to the conditions on the breeding grounds.

We sought to address population shifts in North American mallards by asking the following questions: (1) How do natural and harvest mortality hazard rates vary for different cohorts of mallards breeding in the PPR across both time and space? and (2) How have relationships between environmental conditions and life histories created said variation? We estimated mallard mortality hazard rates and survival in the PPR from 1974 to 2023 using spatially explicit statistical models to examine the spatiotemporal variation in these demographic rates for adult males, adult females, juvenile males, and juvenile females. We also examine potential relationships between natural and harvest mortality and the amount of crop and fallow acreage in the landscape, as well as the relative wet or dryness of an area in a given time.

## 2 Materials and Methods

We estimated cause‐specific mortality hazard rates using release and recovery data for mallards marked with uniquely identifiable metal tarsal bands in the Prairie Pothole Region (1974–2024; USGS Bird Banding Laboratory 2025). We divided the sample into adult males (n = 599,724), adult females (n = 226,727), juvenile males (n = 163,006), and juvenile females (n = 138,632) to examine differences in mortality risk among cohorts. Ducks were classified as local if they were not yet developmentally capable of flight when banded, hatch year if they were hatched in the current year but capable of flight and after hatch year if they were at least one year old. Age was determined using plumage (Krapu et al. 1979). We pooled birds banded as *local* and *hatch year* as juveniles, and individuals banded as *after hatch year* or older as adults. Releases and encounters only included “normal wild birds” without a reward band or markers other than federal‐numbered metal bands. Encounters were subset to include only individuals that were dead at reporting by way of harvest and reported by the finder of the band (a re‐encounter code of HOW = 1, i.e., shot). We generated 100 km × 100 km blocks to create 59 different study cells. This is slightly less than the distance of one degree of latitude (110.574 km) which can capture landscape level processes and can account for some year‐to‐year movement in nesting locations while still demonstrating spatial variation in demographic rates. Moreover, if grid cell sizes were smaller, a greater percentage of grid cells would have parameter estimates interpolated with relatively large credible intervals. This would result in more estimates that have little practical utility.

To understand potential relationships between demographic rates and environmental conditions, we regressed natural mortality hazard rates for all cohorts against the standardized amount of crop and fallow acreage within each cell, and the average Standardized Precipitation‐Evapotranspiration Index (hereafter, SPEI) value from March to August in a given year. SPEI data (48‐month time scale) acted as a proxy for wetland availability and were obtained from SPEIbase (Beguería et al. 2023). Higher values indicate wetter periods and lower values, drier periods. The yearly crop cover and fallow acreage were obtained from the US and Canadian Agriculture Censuses (Census of Agriculture 2022; United States Department of Agriculture 2024). Because both the US and Canadian censuses are conducted only every 5 years, years without data were linearly interpolated using the zoo package in R (Zeileis and Grothendieck 2005). Please see Appendix S1 for figures describing the spatiotemporal variation in these covariates.

### 2.1 Modeling and Computational Details

We modeled the data using an expanded Brownie model (Brownie 1978) to estimate spatially explicit mortality hazard rates via an intrinsic autoregressive model to account for spatial autocorrelation between sites. For a given year (t), a sample of mallards was banded in each cell (s), and subsequent recoveries of birds banded in that cell are recorded. A banded individual fell into one of three categories: (1) it was killed and reported in the same year of banding (j=t), (2) it survived the year and was killed and reported in a later year, or (3) it was never reencountered or reported.

Male and female data were modeled separately as the two sexes had large differences in values for demographic rates, computation efficiency, and individuals banded as juveniles often going on to have adult demographic rates, given they survived past their juvenile year.

A cohort consists of all birds banded in a given study cell s in year t. The observed data were represented by a vector:

$$y_{t,s}={\left(y_{t,s,j}\right)}_{j=t}^{T}$$

where $y_{t,s,j}$ represented the number of birds banded in a cohort (t,s) that were recovered in year j. $y_{t,s,T+1}$ represented all birds from that cohort that were never recovered, where T was the total number of years in the study.

The observed data $y_{t,s}$ followed a multinomial distribution:

$$\begin{array}{r} y_{t,s}∼\text{Multinomial}\left(N_{t,s},p_{t,s}\right) \end{array}$$

Here, $N_{t,s}$ denotes the total number of birds banded in a cohort. The probability vector for recoveries and non‐recoveries is given by $p_{t,s}$.

The probability of never being recovered is given by:

$$\begin{array}{r} p_{t,s,T+1}=1-\sum_{j=t}^{T}p_{t,s,j} \end{array}$$

To be observed in the year of banding (j=t), a bird must be killed by a hunter ($\kappa_{s,t}$), retrieved, or not lost to crippling (1−c), and reported to the Bird Banding Lab ($r_{t}$). All together, these terms constitute an *encounter*. Crippling loss was modeled as a constant equal to 0.2 (Hicklin and Barrow 2004; Zhao et al. 2018). Reporting rates were estimated using priors derived from Boomer et al. (2023). Reporting rates were specific only to year. The probability of recovery in the year of banding is:

$$\begin{array}{r} p_{t,s,t}=\kappa_{s,t}\left(1-c\right)r_{t} \end{array}$$

For recoveries in later years (j>t), the bird must first survive the intervening years. $\phi_{s,m}$ denotes the survival probability from year m to m+1. To survive until year j, the bird must survive each year from t to j−1:

$$\begin{array}{r} \prod_{m=t}^{j-1}\phi_{s,m} \end{array}$$

If the bird survives until the year j, it can then be encountered. Thus, the probability of an encounter in year j>t is:

$$\begin{array}{r} p_{t,s,j}=\left(\prod_{m=t}^{j-1}\phi_{s,m}\right)\kappa_{s,j}\left(1-c\right)r_{j} \end{array}$$

We modeled harvest mortality ($m_{\kappa }$) and natural mortality ($m_{\eta }$) hazard rates as a function of temporal random effects (δ) and spatial random effects (ε), extending (Riecke, Lohman, et al. 2022). Hazard mortality rates are the intensity of mortality events at any given instance. This is opposed to mortality probabilities, which exist in discrete time. We also included independent covariates to explain variation in natural mortality. We used an exponential link function to transform the linear equation:

$$\begin{array}{c} m_{\kappa ,s,t}=\exp\left(\alpha_{\kappa }+\delta_{\kappa ,t}+\varepsilon_{\kappa ,s}\right)\\ m_{\eta ,s,t}=\exp\left(\alpha_{\eta }+\delta_{\eta ,t}+\varepsilon_{\eta ,s}+x_{s,t}^{\prime }\beta \right) \end{array}$$

$x_{s,t}^{\prime }$ is a row vector of covariates measured at site (s) during year t, and α, and β are the corresponding vectors of regression coefficients.

Harvest (κ) and natural mortality (η) probabilities, along with survival (ϕ), were derived as follows:

$$\begin{array}{c} \kappa =1-\exp\left(-m_{k}\right)\\ \eta =\left(1-\kappa \right)\times \left(1-\exp\left(-m_{\eta }\right)\right)\\ \phi =\exp\left(-\left(m_{\kappa }+m_{\eta }\right)\right) \end{array}$$

The use of mortality hazard rates, as opposed to mortality probabilities, allows for modeling to follow the ecological system more closely. Mortality doesn't happen in discrete waves; rather, individuals can die across the year from natural causes or throughout the harvest season at a certain intensity (or mortality hazard rate). Moreover, the use of hazard rates makes the two sources of mortality more easily comparable than the use of mortality probabilities (please see Ergon et al. 2018 for an excellent discussion).

The spatial random effects $\varepsilon_{\kappa ,s}$ and $\varepsilon_{\eta ,s}$ follow a multivariate normal distribution with a mean vector of zero and a covariance structure governed by an intrinsic conditional autoregressive (ICAR) model:

$$\begin{array}{c} \varepsilon_{\kappa }∼\mathrm{MVN}\left(0,\Sigma_{\kappa }\right),\\ \varepsilon_{\eta }∼\mathrm{MVN}\left(0,\Sigma_{\eta }\right) \end{array}$$

The covariance matrices $\Sigma_{\kappa }$ and $\Sigma_{\eta }$ are given by:

$$\begin{array}{c} \Sigma_{\kappa }=v_{\kappa }^{2}{\left(D-W\right)}^{-1},\\ \Sigma_{\eta }=v_{\eta }^{2}{\left(D-W\right)}^{-1} \end{array}$$

D is a diagonal matrix with the number of neighbors for each site along the diagonal and W is a spatial adjacency matrix defining neighborhood relationships. The variance terms $v_{\eta }^{2}$ and $v_{\kappa }^{2}$ control the overall magnitude of spatial variation and were modeled with a vague prior, $v∼\text{Gamma}\;\left(1,1\right)$. All other priors and modeling details can be found in Appendix S2.

It is important to note that this model assumes birds return to their initial banding grid cell each year. While this assumption is certainly violated, previous research has shown that mallards have relatively high fidelity to the broader landscapes they breed within, particularly adults and females (Doherty et al. 2002; Cunningham et al. 2016), which 100 km^2^ would largely encompass.

With this as our basic model, we increased computational efficiency by using a sparse ICAR model, as detailed in (Jin et al. 2005). This model was fit in Stan (Carpenter et al. 2017) for 3500 iterations. Traceplots and Gelman‐Rubin diagnostics indicated convergence (averaging 1.0004 for the female only model and 1.0009 for the male only model across all parameters; Gelman and Rubin 1992). Finally, we also fit a non‐spatial model using the same data and covariates pooled across the entirety of the landscape to serve as a point of comparison for our spatially explicit approach (Appendix S4).

## 3 Results

### 3.1 Survival

Survival varied substantially in space and time for all cohorts, but tended to be highest in the central portion of the region for all cohorts (Figure 1). Mean adult female survival tended to be lowest in the eastern Dakotas and highest in the northern Canadian prairies. Similarly, mean juvenile female survival was high in the northern Canadian prairies. Spatial variation in survival was greater for juvenile cohorts than either female or male adults. Critically, areas of high survival moved in response to changes in spatiotemporal covariates. For instance, in the mid‐1990s through mid‐2000s, survival was highest in the northern Canadian prairies. However, beginning in the late 2000s, high survival rates shifted towards the southeast for all cohorts (Figure 2). Adult and juvenile female survival declined in the latter half of the time series, with decreases in survival beginning in the late 1980s (Figure 3). This trend was particularly pronounced for juvenile females and sharply contrasted with increases in juvenile male survival. Adult males had no distinct trend in survival rate throughout the study period relative to all other cohorts.

**FIGURE 1:**
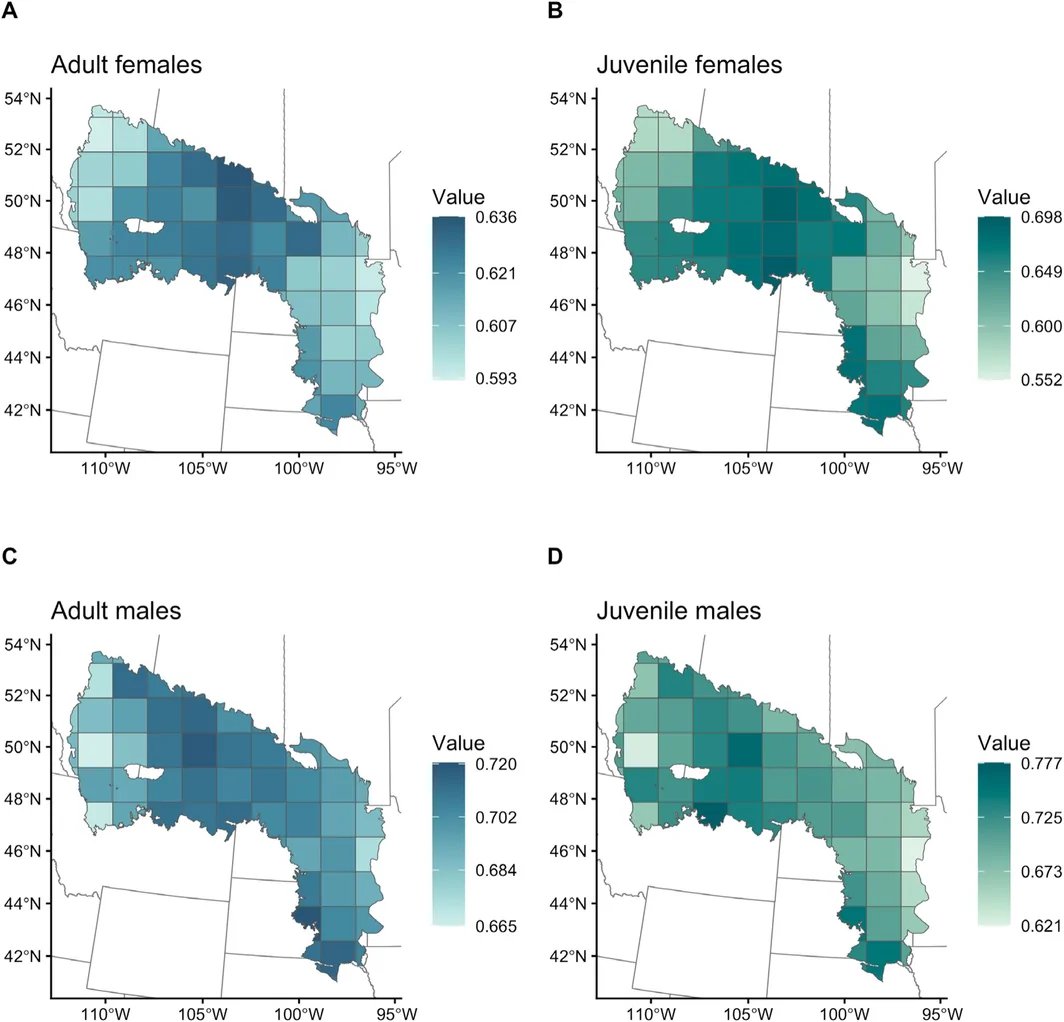
Mean survival of adult female (A), juvenile female (B), adult male (C), and juvenile male (D) mallards in the Prairie Pothole Region from 1974 to 2023. Darker shades represent higher values.

**FIGURE 2:**
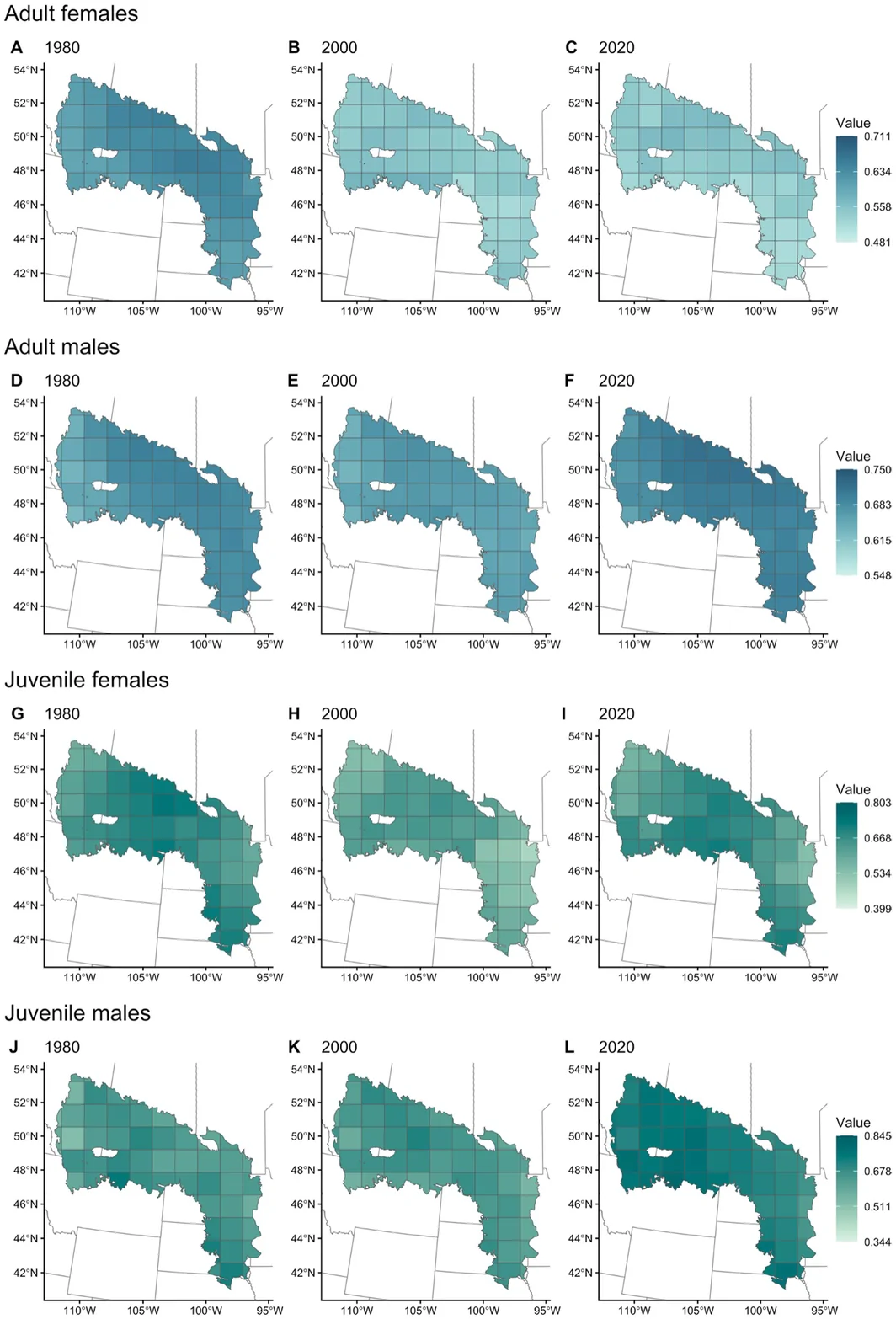
Survival in the Prairie Pothole Region in the years 1980 (A, D, G, J), 2000 (B, E, H, K), and 2020 (C, F, I, L).

**FIGURE 3:**
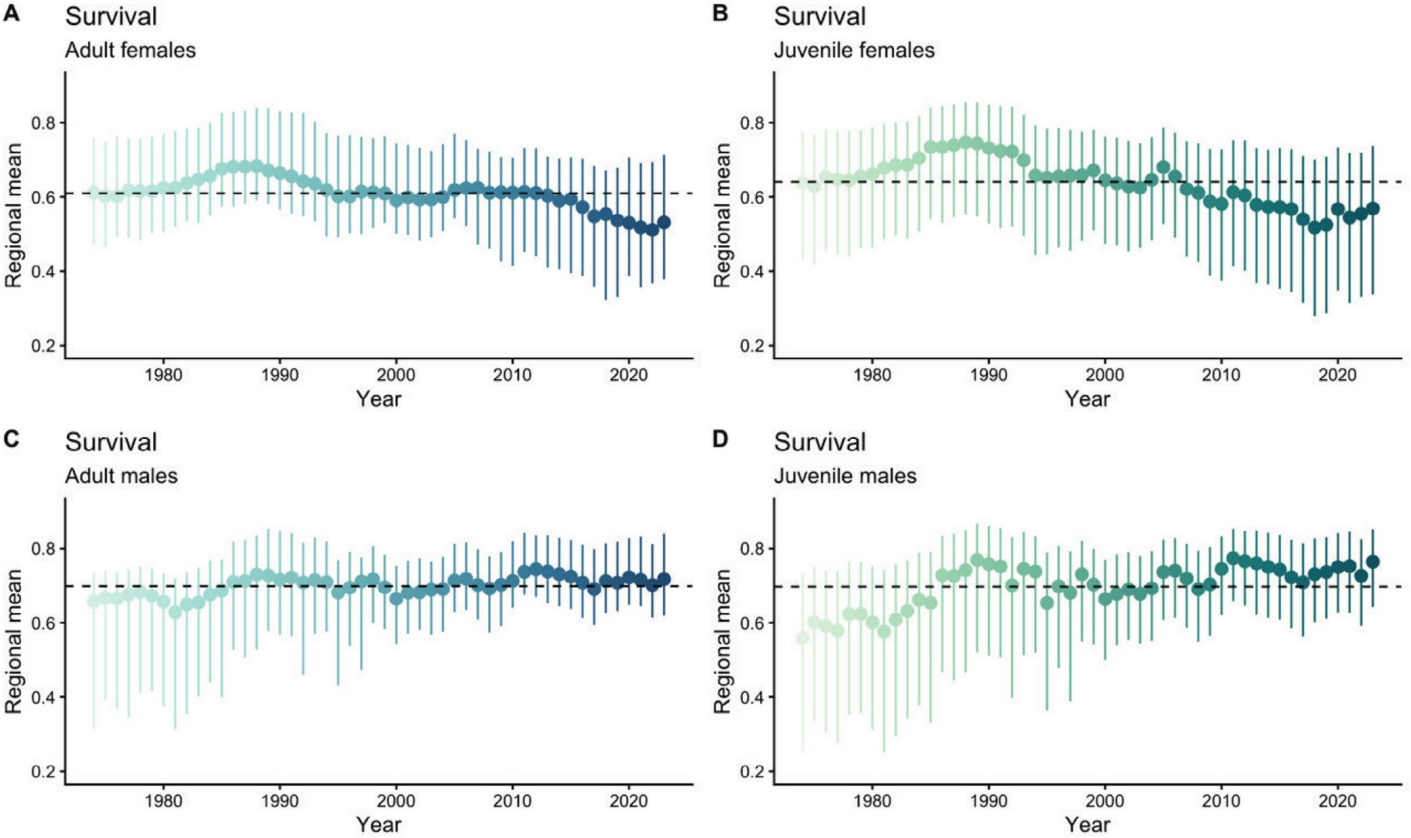
Medians and 95% credible intervals for estimates of survival across the Prairie Pothole Region from 1974 to 2023 for adult female (A), juvenile female (B), adult male (C), and juvenile male (D) mallards aggregated across sites. The dotted line represents the average survival rate across the time period.

### 3.2 Harvest Mortality

Harvest mortality showed clear spatial and temporal trends for all cohorts. Harvest mortality tended to be highest for birds banded in the eastern portion of the region for all cohorts (Appendix S3, Figures S3.1 and S3.2). This spatial trend was consistent throughout the time period. Although harvest mortality for adults tended to be less variable than juvenile harvest mortality, harvest mortality rates showed substantial shifts throughout time for all cohorts, and these changes were temporally synchronous (Figure 4; Appendix S3, Figure S3.2). For instance, harvest mortality was particularly low for all classes in the late 1980s and early 1990s during a major drought and a period of restrictive harvest regulations, but increased beginning in the early 1990s and then dipped again in the early to mid 2000s (Appendix S3, Figure S3.2). Juvenile harvest mortality was higher than adult harvest mortality throughout the time period, and males in both age classes were harvested at higher rates relative to females.

**FIGURE 4:**
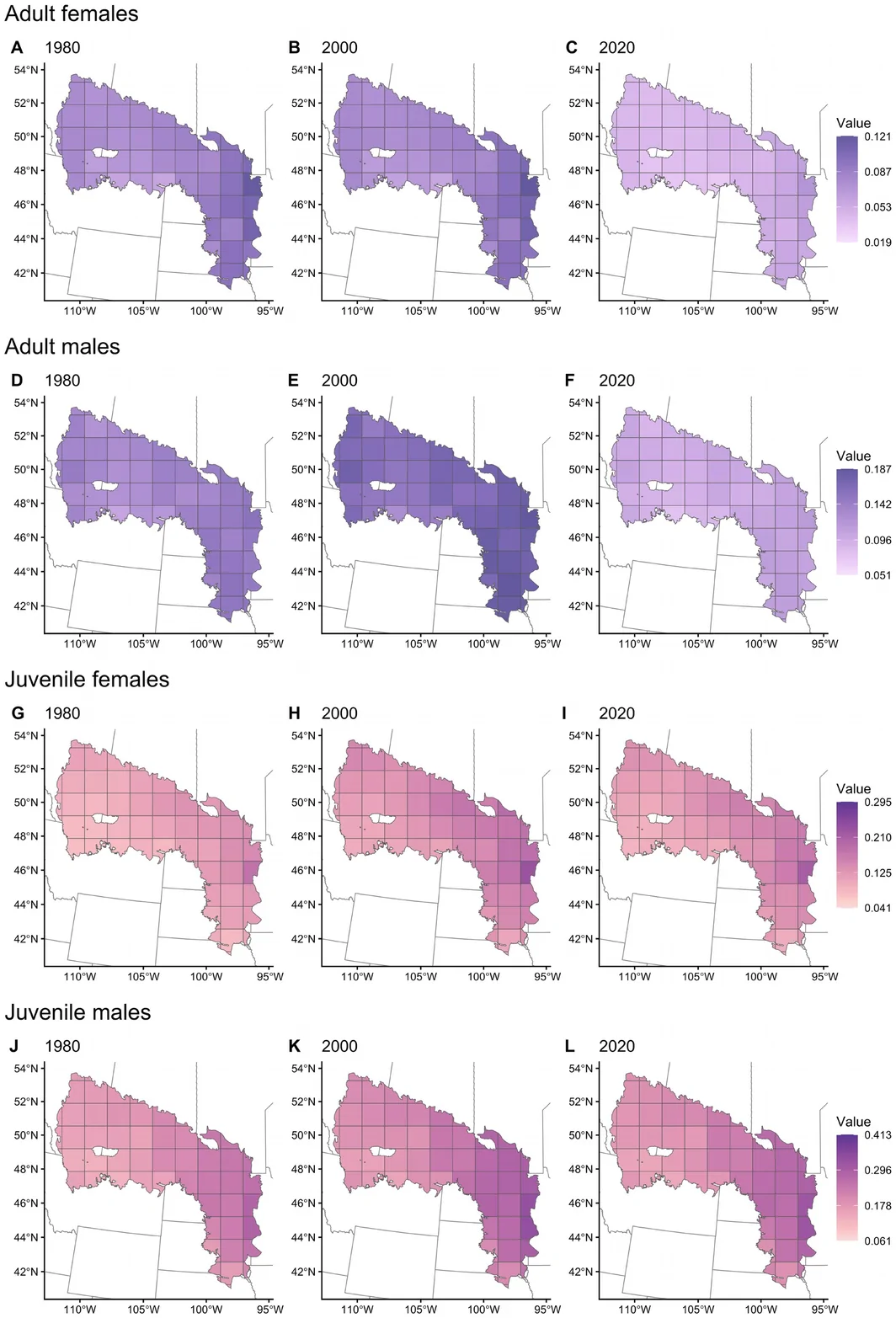
Harvest mortality in the Prairie Pothole Region in the years 1980 (A, D, G, J), 2000 (B, E, H, K), and 2020 (C, F, I, L).

### 3.3 Natural Mortality

Natural mortality among males was consistently highest in the western portion of the PPR (Appendix S3, Figure S3.3). Adult female natural mortality was consistently higher than other cohorts, and there was substantially more spatiotemporal variation of adult female natural mortality (Figures 5 and 6). Temporally, male natural mortality slightly declined and seemed to stabilize by the end of the time period. In contrast, female natural mortality increased and became increasingly variable after the 1990s (Figures 5 and 6). For instance, adult female mortality increased after around 2010, especially in the SE portion of the PPR. Adult males, in contrast, did not show spatial shifts to the same magnitude and temporally, their natural mortality has declined (Figure 5).

**FIGURE 5:**
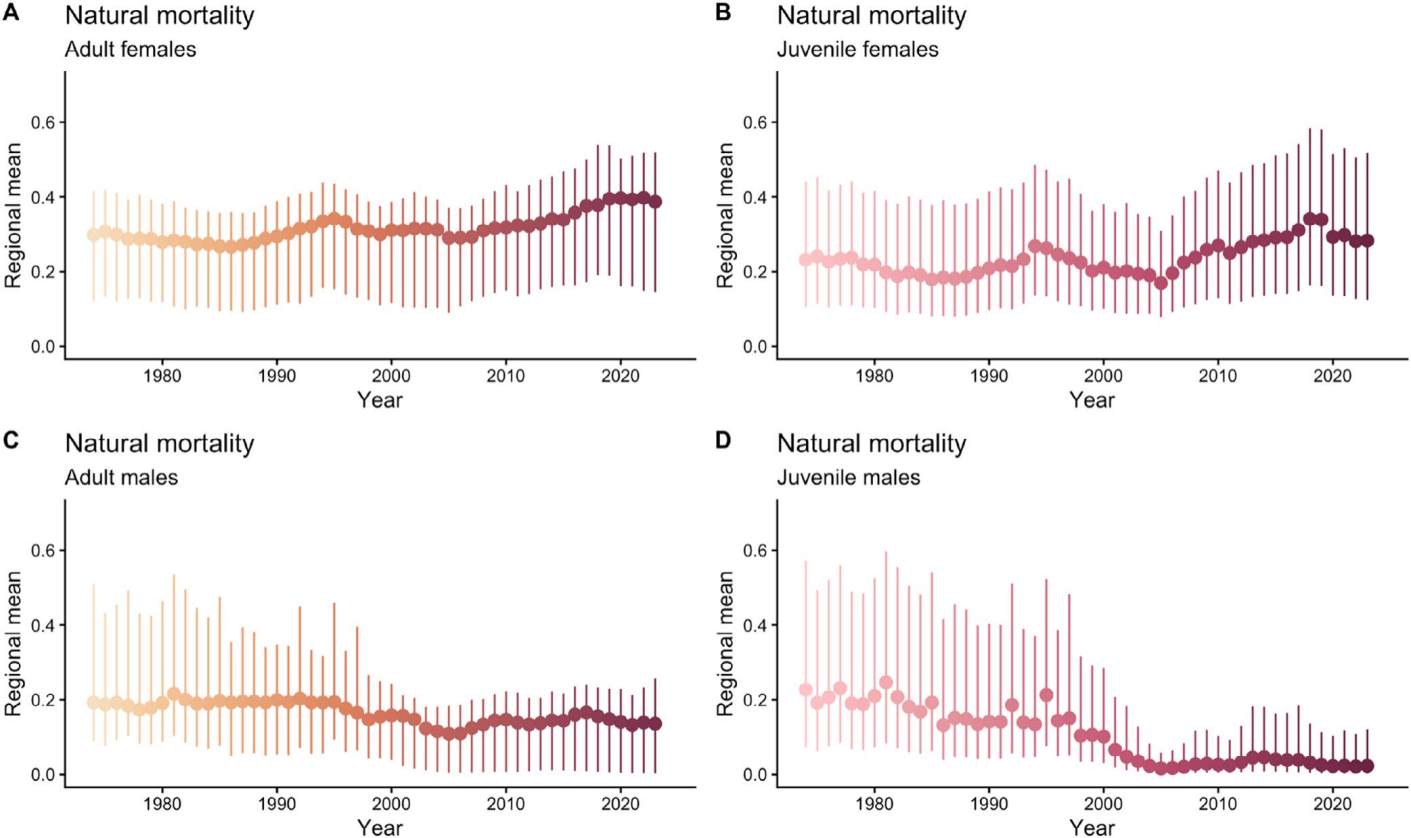
Medians and 95% credible intervals for estimates of natural mortality across the Prairie Pothole Region from 1974 to 2023 for adult female (A), juvenile female (B), adult male (C), and juvenile male (D) mallards.

**FIGURE 6:**
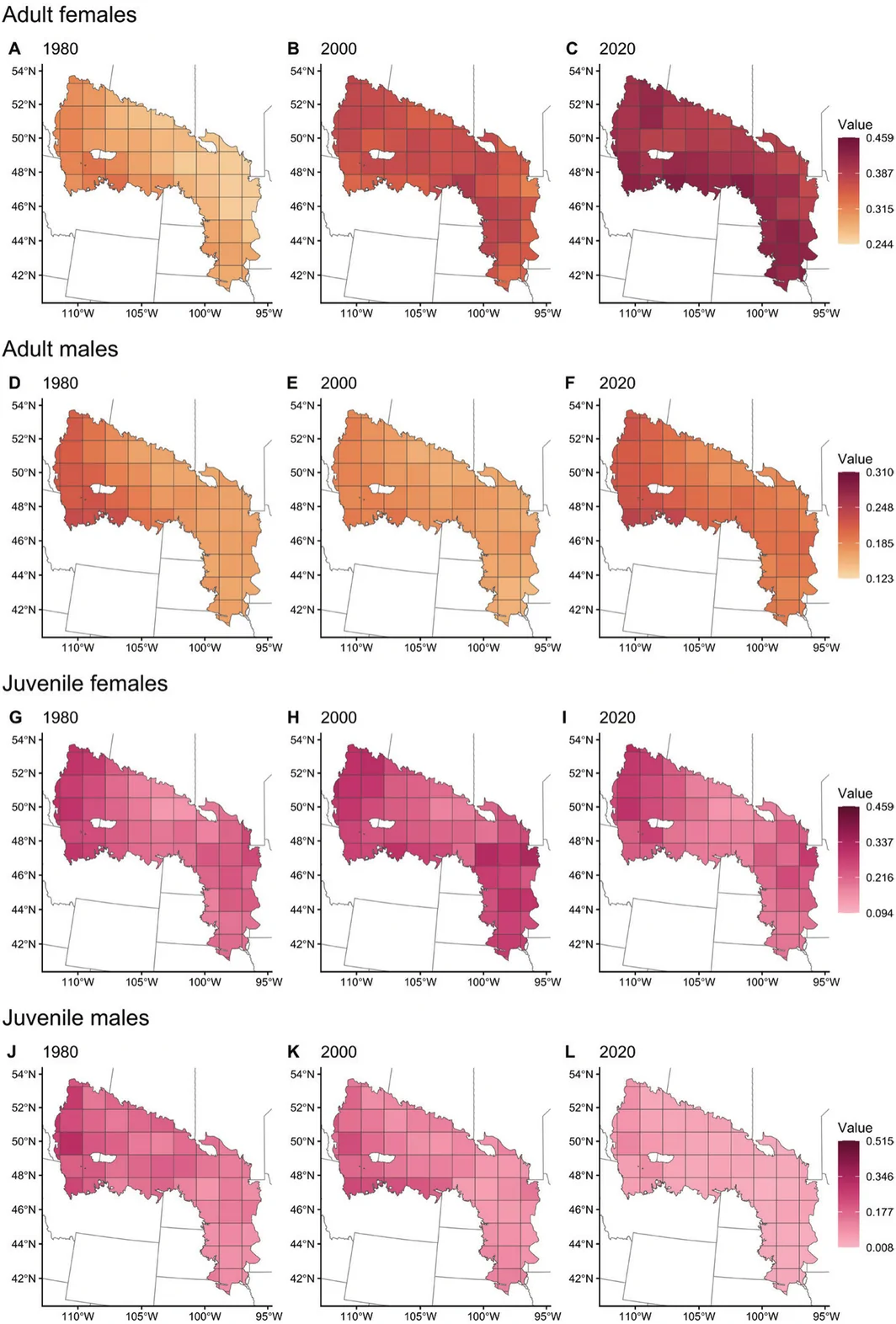
Natural mortality in the Prairie Pothole Region in the years 1980 (A, D, G, J), 2000 (B, E, H, K), and 2020 (C, F, I, L).

### 3.4 Relationships With Spatiotemporal Covariates

Adult female natural mortality had a negative relationship with crop and fallow acreage ($\beta_{\text{crop}}$ = −0.05, 95% CRI: −0.08 to −0.02; $\beta_{\text{fallow}}$ = −0.04, 95% CRI: −0.07 to −0.02) and a positive correlation with SPEI ($\beta_{\text{SPEI}}$ = 0.07, 95% CRI: 0.04–0.11). Adult male natural mortality did not have a strong relationship with crop and fallow acreage but was positively correlated with SPEI ($\beta_{\text{SPEI}}$ = 0.07, 95% CRI: 0.04–0.08). In contrast to adult age classes, crop acreage had a positive relationship with juvenile female ($\beta_{\text{crop}}$ = 0.19, 95% CRI: 0.04–0.35) and male ($\beta_{\text{crop}}$ = 0.3, 95% CRI: 0.13–0.47) natural mortality. Juvenile male natural mortality also had a positive association with fallow acreage ($\beta_{\text{fallow}}$ = 0.22, 95% CRI: 0.08–0.35). See Figure 7 for a complete overview of the regression coefficients associated with natural mortality estimates. When this model was fit without the spatial terms so that only temporal autocorrelation was accounted for, the relationships between natural mortality and habitat variables were obscured, particularly for females (Appendix S4, Figure S4.1). In the non‐spatial model, habitat data were pooled across the whole of the PPR, but most banding occurred in only a few areas. Thus, region‐wide habitat trends were regressed against demographic rates estimated from data clustered in specific, smaller areas. This served to mask some of the regional variation in habitat variables and confound relationships between the demographic rates and covariates.

**FIGURE 7:**
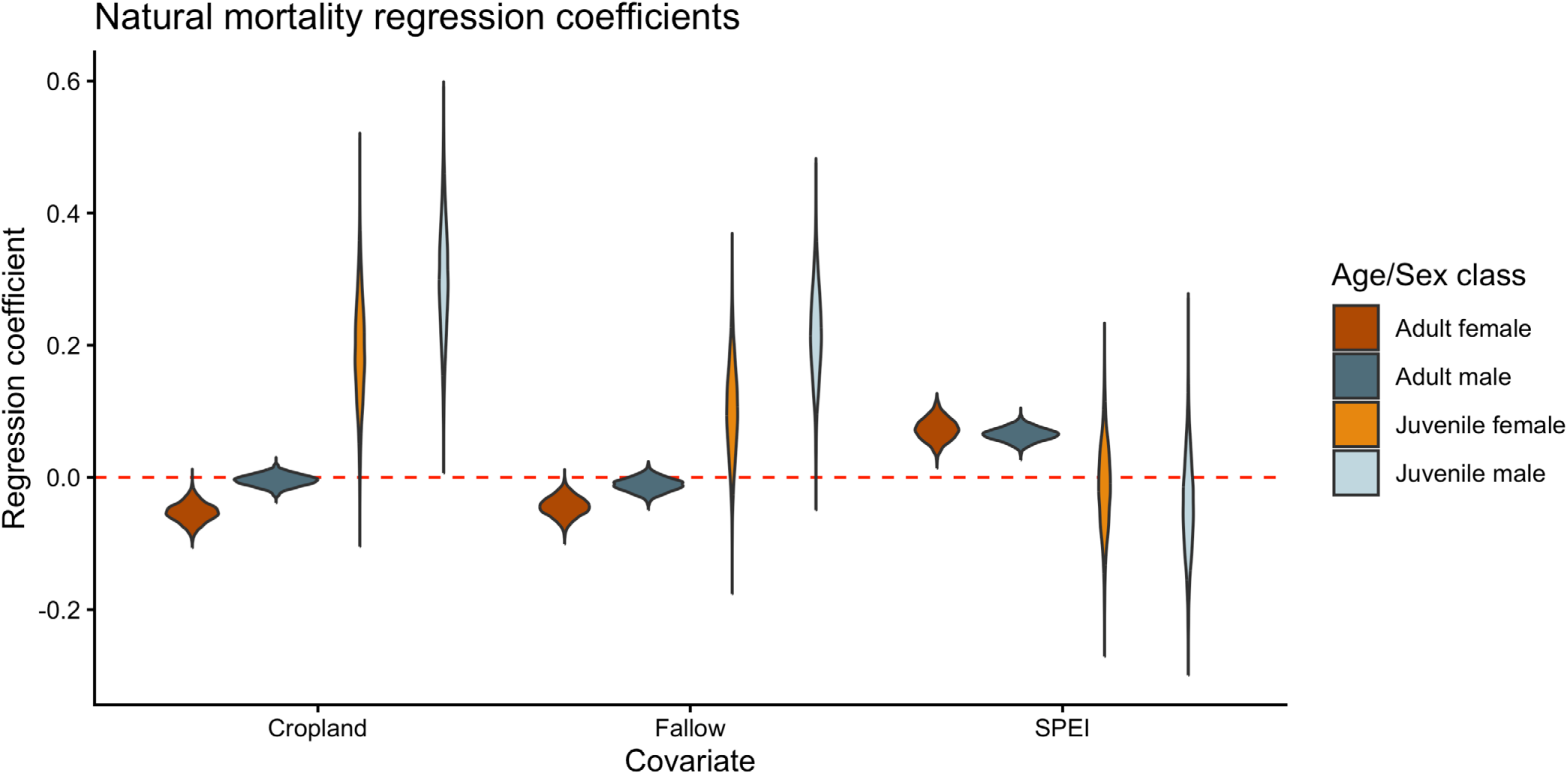
Violin plots representing the posterior distribution of regression coefficients associated with natural mortality hazard rates for adult female (dark orange), adult male (dark blue), juvenile female (light orange), and juvenile male (light blue) mallards in the Prairie Pothole Region from 1974 to 2023.

## 4 Discussion

### 4.1 Adult Female Survival Is Driven by Natural Mortality

Annual survival of adult female mallards varied substantially throughout both space and time. In general, it tended to be highest in the northern PPR but declined across the study region from 1980 to 2020. These patterns were driven by increases in natural mortality through time, especially in the southeastern and extreme northwestern PPR. Trends in natural mortality were associated with changes in both environmental conditions and agricultural practices.

Natural mortality increased and became more variable over time for females, particularly after the mid‐1990s, but seemed to decrease and then stabilize for males over the study period. However, female natural mortality showed more distinct spatiotemporal patterns than male natural mortality, and female natural mortality was more strongly associated with spatiotemporally varying environmental conditions, as expected. Environmental conditions on breeding grounds disproportionately affect breeding females, as they face higher risks and costs associated with reproduction (Arnold and Clark 1996; Krapu et al. 1983; Riecke, Sedinger, et al. 2022; Singer et al. 2016; Van Noordwijk and de Jong 1986).

Increased wetness was positively related to adult natural mortality but had no relationship with juvenile natural mortality. Increased numbers of ponds and wetlands are associated with higher nest and brood survival (Singer et al. 2016; Walker et al. 2013). Individuals tend to have higher reproductive effort (Krapu et al. 1983; Singer et al. 2016; Van Noordwijk and de Jong 1986) and face more risks associated with reproducing (Arnold and Clark 1996), even though favorable environmental conditions result in higher recruitment. Adults that invest more energy in reproduction may have more difficulty surviving the breeding season (Van Noordwijk and de Jong 1986). Put simply, environmental conditions associated with higher fecundity were related to higher adult natural mortality.

Particularly on the part of females, increased reproductive investment in favorable years can lead to increased predation risk while incubating (Arnold et al. 2012; Devries et al. 2003) and lower nutritional condition leading to mortality after the breeding season (Rotella et al. 2003). These effects can then result in an increase in the natural mortality rate (Dufour et al. 1993). Adult females tend to nest and re‐nest at lower rates during drought periods as fewer resources are available to sustain the female through egg production, incubation, and brood rearing (Dufour and Clark 2002; Krapu et al. 1983). This pattern is consistent with the demographic buffering hypothesis, since adults invest less in reproduction during a breeding season with unfavorable environmental conditions that could potentially allow for higher reproductive output in subsequent years when conditions are more favorable (Van Noordwijk and de Jong 1986).

Higher proportions of cropland were associated with higher juvenile natural mortality, but had only a small, negative relationship with adult female natural mortality. Cropland is poor habitat for nesting and raising broods (Bethke and Nudds 1995; Devries et al. 2023; Drever et al. 2007; Higgins 1977). These areas tend to have high levels of agricultural disturbance, leading to low nest success through nest destruction or predation (Cowardin et al. 1985; Devries et al. 2023; Drever et al. 2007; Greenwood et al. 1995; Rischette et al. 2021). Predation on hens is also generally higher in cropland because of high predator concentrations in the surrounding area (Greenwood et al. 1995). Moreover, increases in cropland acreage in active use can reduce other types of habitats that females may prefer, such as grassland or idle agricultural fields (Greenwood et al. 1995; Rischette et al. 2021). This may lead to reduced nesting effort on the part of females (Rischette et al. 2021). As a result of these factors, mallards tend to nest at lower rates in agricultural fields (Rischette et al. 2021). Females may put less effort into reproduction when landscapes are dominated by croplands, thus reducing their natural mortality risk (Devries et al. 2003).

In contrast, the natural mortality of juveniles depends in part on the quality of their habitat during growth and development (Cox Jr et al. 1998). Habitats, including nearby wetlands and ponds, may be of poorer quality when enmeshed in an agricultural landscape (Kraus et al. 2021). Agricultural runoff can limit the growth of invertebrate populations and wetland vegetation (Wrubleski and Ross 2011). This may limit the growth of ducklings in these areas (Sjöberg et al. 2000) and thus increase their natural mortality rates via carry‐over effects of conditions during growth (Anderson 1980; Cox Jr et al. 1998).

Fallow acreage had a positive relationship with only juvenile male natural mortality and a small negative correlation with adult female natural mortality. Fallow land may serve as a signal of less intensive agriculture (Weegman et al. 2024). However, it may exist within a larger cropland matrix and does not always provide excellent nesting cover or adequate resources for nesting and incubation (Devries et al. 2023). Adult females may thus reduce nesting effort in these habitats (Devries et al. 2023). However, given the slight long‐term increase in adult female natural mortality and small decrease in juvenile male natural mortality, along with a decrease in overall fallow acreage, the relationships estimated here may simply arise from temporal trends changing in parallel, rendering the relationships spurious.

Harvest mortality increased over time, especially after the 1990s. In general, harvest mortality tended to be highest for individuals marked in the SE portion of the study area and consistently higher for males, as compared to females. Adult females consistently had the lowest harvest mortality rate out of any cohort.

The temporal trend documented here is particularly evident in juvenile females and juvenile males, suggesting that younger birds experienced increasing pressure from harvest mortality as time progressed. Previous studies have shown that juveniles are more vulnerable to harvest (Alisauskas et al. 2014; Francis et al. 1992). This is often attributed to naïveté about hunting practices such as decoys (Giroux and Bédard 1986). The temporal patterns we observed could reflect changes in hunting regulations and hunting techniques that affect juveniles more than adults. For example, spinning wing decoys have a greater effect on juveniles relative to adults (Szymanski and Afton 2005). The emergence and legalization of this technology may influence variation in juvenile harvest mortality to a greater extent than that of adults.

Overall, these results indicate that natural mortality, rather than harvest mortality, drove trends in adult survival, particularly for females. They also align with previous research indicating that recruitment, rather than adult survival, drives most of the changes in the dynamics of the midcontinent mallard population (Hoekman et al. 2002; Coluccy et al. 2008; Koons et al. 2014; Specht and Arnold 2018). It is notable that the areas of highest female natural mortality occur where population growth is estimated to be highest, in the SE of the PPR (Janke et al. 2017).

### 4.2 Male and Female Survival Are Increasingly Diverging

Survival of both adult and juvenile females declined beginning in the late 1980s as natural mortality increased (Figure 6). Adult male survival was relatively constant, and juvenile male survival rates increased throughout the time period (Figure 5). This divergence in survival resulted in a long‐term increase in the difference between the survival rates of males and females. Sex‐specific survival differences may be related to the increased costs of reproduction for females relative to males (Romano et al. 2022; Székely et al. 2014; Wood et al. 2021). Increased mortality risk associated with predation during nest incubation (Arnold et al. 2012; Devries et al. 2003), re‐nesting (Dufour and Clark 2002), or other energetic costs associated with reproduction on the breeding grounds (Rotella et al. 2003) may be driving this trend. Long‐term changes in the difference between male and female survival may also lead to an increasingly male‐biased sex ratio for this population (Alisauskas et al. 2014). In many waterfowl species, sex ratios at hatch are even (Blums and Mednis 1996; Donald 2007; Lemons et al. 2012). Thus, diverging survival rates between males and females necessitate changes in adult sex ratios for the population. If sex ratios become too male‐skewed, the population may shrink as breeding females limit population growth (Eberhart‐Phillips et al. 2017). These results raise concerns about the ability of recruitment rates to outweigh the loss of females and maintain current population sizes. Researchers should examine the potential for skewed sex ratios to affect population growth rates and project these consequences into the future.

### 4.3 Conservation Implications

These findings emphasize the importance of habitat conservation and restoration to support mallard populations, particularly in light of changing environmental conditions. Long‐term changes in land use and climate have led to clear changes in mallard demographic rates underlying observed changes in mallard distribution and population growth (Janke et al. 2017). Efforts to conserve existing habitat, including increasing the amount of land with conservation easements, decreasing the intensity of agriculture, and protecting native grasslands in both Canada and the US, may help buffer future population declines (Zhao et al. 2019; Buderman et al. 2020). In particular, it is critical that researchers determine the causes of changes in female mortality rates. It is also worth noting clear trade‐offs between fecundity and survival. Lower quality breeding habitats (e.g., areas with large amounts of intensive agriculture) actually increase female survival by decreasing reproductive investment. However, high‐quality conditions on breeding grounds are necessary for stable mallard populations to maintain adequate recruitment.

## 5 Conclusion

The observed shifts in survival, natural mortality, and harvest mortality underscore the complex interplay between life history traits and environmental conditions. Of particular concern is the declining survival of female mallards, which has significant implications for this population. Understanding the underlying mechanisms driving these demographic shifts is essential for targeting conservation efforts and designing management strategies. By identifying critical habitats and environmental conditions that influence demographic rates, managers can develop more effective approaches to maintaining mallard populations in the face of environmental change. Furthermore, the spatially explicit model that we demonstrate herein recovered parameter estimates that differed substantially from a non‐spatial model. We suggest that spatially explicit models may serve to improve inference for long‐term landscape‐scale demographic studies. This research supports past work (Janke et al. 2017), demonstrating that the habitat shifts in the PPR are generating substantial shifts in waterfowl demography across space and time.

## Author Contributions

**Madeleine G. Lohman:** conceptualization (equal), formal analysis (equal), funding acquisition (equal), methodology (equal), visualization (equal), writing – original draft (equal), writing – review and editing (equal). **Thomas V. Riecke:** conceptualization (equal), funding acquisition (equal), methodology (equal), writing – review and editing (equal). **James S. Sedinger:** supervision (equal), writing – review and editing (equal). **Perry J. Williams:** methodology (equal), supervision (equal), writing – review and editing (equal).

## Funding

This work was supported by the National Science Foundation Graduate Research Fellowship Program and the Institute for Wetland and Waterfowl Research, Ducks Unlimited Canada.

## Disclosure

Statement on Inclusion: Our study fails to include scientists based in one of the countries (Canada) where the study was conducted. The other country (United States) is represented by all authors. We recognize that more could have been done to engage local scientists with our research as our project developed, and to embed our research within the national context and research priorities. We plan to address these issues in future research.

## Conflicts of Interest

The authors declare no conflicts of interest.

## Supporting information


**Figure S3.1:** Mean harvest mortality of adult female (A), juvenile female (B), adult male (C), and juvenile male (D) mallards in the Prairie Pothole Region from 1974 to 2024. Darker shades represent higher values.
**Figure S3.2:** Mean harvest mortality across the Prairie Pothole Region from 1974 to 2024 for adult female (A), juvenile female (B), adult male (C), and juvenile male (D) mallards.
**Figure S3.3:** Mean natural mortality of adult female (A), juvenile female (B), adult male (C), and juvenile male (D) mallards in the Prairie Pothole Region from 1974 to 2023. Darker shades represent higher values.
**Figure S4.1:** Mean natural mortality across the Prairie Pothole Region from 1974 to 2023 for adult female (A), juvenile female (B), adult male (C), and juvenile male (D) mallards from models not incorporating spatial variation.
**Figure S4.2:** Mean harvest mortality across the Prairie Pothole Region from 1974 to 2023 for adult female (A), juvenile female (B), adult male (C), and juvenile male (D) mallards from models not incorporating spatial variation.
**Figure S4.3:** Mean survival across the Prairie Pothole Region from 1974 to 2023 for adult female (A), juvenile female (B), adult male (C), and juvenile male (D) mallards from models not incorporating spatial variation.
**Figure S4.4:** Violin plots representing the posterior distribution of regression coefficients associated with natural mortality hazard rates for adult female (dark orange), adult male (dark blue), juvenile female (light orange), and juvenile male (light blue) mallards in the Prairie Pothole Region from 1974 to 2023. These regression coefficients come from the model not incorporating spatial variation.

## Data Availability

Data and modeling files have been uploaded to Dryad (DOI: 10.5061/dryad.rfj6g57sg).
