# Supplementary material for: Climate and Other Human Causes of Spatial Variation in Mallard Mortality and Population Dynamics
Source: Ecol Evol. 2026 Oct 29;16(11):e74596. doi: 10.1002/ece3.74596 (PMC13719240; doi:10.1002/ece3.74596)
Supplement: Supplementary file 1 [file ECE3-16-e74596-s001.docx]

# Appendix 1

GIF files showing the spatiotemporal variation in covariates and number of releases for each grid cell can be found at https://github.com/madeleinelohman/spatial_mallard.

# Appendix 2

The temporal random effects $\eta_{t}$ and $\epsilon_{t}$ for $t=2,\ldots,T$ follow autoregressive normal priors:

$$\begin{aligned} \eta_{t}&\sim\text{Normal}(\eta_{t-1},\sigma_{\kappa}^{2}), t=2,\ldots,T, \\ \epsilon_{t}&\sim\text{Normal}(\epsilon_{t-1},\sigma_{\phi}^{2}), t=2,\ldots,T. \end{aligned}$$

For the initial temporal random effects at $t=1$, the priors are centered at zero:

$$\begin{aligned} \eta_{1}&\sim\text{Normal}(0,\sigma_{\kappa}^{2}), \\ \epsilon_{1}&\sim\text{Normal}(0,\sigma_{\phi}^{2}). \end{aligned}$$

Finally, the variance parameters $\sigma_{\kappa}$ and $\sigma_{\phi}$ are assigned uniform priors:

$$\begin{aligned} \sigma_{\kappa},\sigma_{\phi}&\sim\text{Uniform}(0,5). \end{aligned}$$

Regression coefficients ($\beta$) all have the following priors:

$$\begin{matrix} \boldsymbol{\beta}\sim\text{Normal}(0,2.25). \end{matrix}$$

Priors for reporting rates were as follows, where α_t_ and β_t_ were moment matched values from reporting rates estimated by Boomer et al. (2023*)*:

$$r_{t}\sim\text{Beta}\left( \alpha_{t},\beta_{t} \right).$$

Parameters associated with harvest and natural mortality estimates are derived from Riecke et al. (2022b). Priors associated with females are:

$$\begin{aligned} \alpha_{hm}^{adult}&\sim\text{Normal}(-0.9,1) \\ \alpha_{hm}^{juvenile}&\sim\text{Normal}(-1.3,1) \\ \alpha_{nm}^{adult}&\sim\text{Normal}(-0.8,1) \\ \alpha_{nm}^{juvenile}&\sim\text{Normal}(-1.2,1) \end{aligned}$$

Priors associated with males are:

$$\begin{aligned} \alpha_{hm}^{adult}&\sim\text{Normal}(-1.6,1) \\ \alpha_{hm}^{juvenile}&\sim\text{Normal}(-1.6,1) \\ \alpha_{nm}^{adult}&\sim\text{No}\text{rmal}(-1.5,1) \\ \alpha_{nm}^{juvenile}&\sim\text{Normal}(-1.5,1) \\ \end{aligned}$$

**Juvenile demographic rates** Models for juvenile demographic rates and probabilities are nearly identical to the adult model, but include parameters accounting for *juvenile survival probability* ($\phi^{*}$), *juvenile natural mortality rate* ($m_{\eta}^{*}$)and *probability* ($\eta^{*}$), and *juvenile harvest mortality rate* ($m_{\kappa}^{*}$)and *probability* ($\kappa^{*}$).

Juvenile demographic probabilities can be derived from demographic rates as follows:

$$\begin{aligned} \boldsymbol{\kappa}^{\mathbf{*}}&=1-e^{-\boldsymbol{m}_{\boldsymbol{\kappa}}^{\mathbf{*}}} \\ \boldsymbol{\eta}^{\mathbf{*}}&=(1-\boldsymbol{\kappa}^{\mathbf{*}})\times\left( 1-e^{-\boldsymbol{m}_{\boldsymbol{\eta}}^{\mathbf{*}}} \right) \\ \boldsymbol{\phi}^{\mathbf{*}}&=exp\left( -(\boldsymbol{m}_{\boldsymbol{\kappa}}^{\mathbf{*}}+\boldsymbol{m}_{\boldsymbol{\eta}}^{\mathbf{*}}) \right). \end{aligned}$$

The probability for a juvenile to be killed, recovered, and reported the same year as banding is:

$$\begin{matrix} p_{t,s,t}^{*}=\kappa_{s,t}^{*}(1-c)r_{t}. \end{matrix}$$

Recovery during the harvest season following the first year of life can be modeled as follows:

$$\begin{matrix} p_{t,s,t+1}^{*}=\left( \phi_{s,t}^{*} \right)\kappa_{s,j}(1-c)r_{t}. \end{matrix}$$

Because juveniles take on adult demographic probabilities beginning the summer after hatch, subsequent probabilities in the juvenile probability simplex ($p^{*}$) must include the juvenile survival probability for the year of banding ($t$) and then the adult survival and harvest mortality probabilities. All subsequent recoveries of individuals can be modeled as follows:

$$\begin{matrix} p_{t,s,j}^{*}=\left( \phi_{s,t}^{*}\prod_{m=t+1}^{j-1} \phi_{s,m} \right)\kappa_{s,j}(1-c)r_{t}. \end{matrix}$$

As in adults, the probability of never being recovered is simply:

$$\begin{matrix} p_{t,s,\emptyset}^{*}=1-\sum_{j=t}^{T} p_{t,s,j}^{*}. \end{matrix}$$

Priors on juvenile survival, harvest mortality, variances, and regression coefficients are the same as those for the adult age class.

# Appendix 3

Additional figures associated with natural mortality and harvest mortality probabilities.

**Figure 3.1.** Mean harvest mortality of adult female (**A**), juvenile female (**B**), adult male (**C**), and juvenile male (**D**) mallards in the Prairie Pothole Region from 1974 - 2024. Darker shades represent higher values.

**Figure 3.2.** Mean harvest mortality across the Prairie Pothole Region from 1974 - 2024 for adult female (**A**), juvenile female (**B**), adult male (**C**), and juvenile male (**D**) mallards.

**Figure 3.3** Mean natural mortality of adult female (**A**), juvenile female (**B**), adult male (**C**), and juvenile male (**D**) mallards in the Prairie Pothole Region from 1974 - 2023. Darker shades represent higher values.

# Appendix 4

## Non-spatial modeling details

The non-spatial model follows the same structure as the model listed within the main manuscript. The non-spatial model excludes the CAR prior on the covariance matrix and instead uses separate variances for the untransformed harvest mortality ($\eta$) and survival ($\epsilon$) rates. The temporal variances are denoted as $\sigma_{\phi}$ for survival and $\sigma_{\kappa}$ for harvest mortality. Spatial variances are shown as $\upsilon_{\phi}$ and $\upsilon_{\kappa}$ for survival and harvest mortality, respectively. Survival ($\phi$) and harvest mortality ($\kappa$) are modeled as listed in the manuscript for the CAR model. The only differences involve the spatial structure on the spatial random effect. These changes can be seen in the following:

$$\begin{aligned} \sigma_{\phi}^{2}&\sim\text{Uniform}(0,5) \\ \sigma_{\kappa}^{2}&\sim\text{Uniform}(0,5) \\ \\ \upsilon_{\phi}&\sim\text{Normal}(0,5) \\ \upsilon_{\kappa}&\sim\text{Uniform}(0,5) \\ \\ \varepsilon_{\phi,s}&\sim\text{Normal}(0,\sigma_{\phi}^{2}) \\ \varepsilon_{\kappa,s}&\sim\text{Normal}(0,\sigma_{\kappa}^{2}) \\ \\ \eta_{t=1}&\sim\text{Normal}(0,\sigma_{\phi}^{2}) \\ \epsilon_{t=1}&\sim\text{Normal}(0,\sigma_{\kappa}^{2}) \\ \\ \eta_{t}&\sim\text{Normal}(\eta_{t-1},\sigma_{\phi}^{2}) \\ \epsilon_{t}&\sim\text{Normal}(\epsilon_{t-1},\sigma_{\kappa}^{2}) \\ \\ \text{logit}(\kappa_{t,s})&=\boldsymbol{X\beta}+\eta_{t}+\varepsilon_{\kappa,s} \\ \text{logit}(\phi_{t,s})&=\boldsymbol{X\alpha}+\epsilon_{t}+\varepsilon_{\phi,s}. \end{aligned}$$

## Demographic rates estimated from the non-spatial model

**Figure 4.1.** Mean natural mortality across the Prairie Pothole Region from 1974 - 2023 for adult female (**A**), juvenile female (**B**), adult male (**C**), and juvenile male (**D**) mallards from models not incorporating spatial variation.

**Figure 4.2.** Mean harvest mortality across the Prairie Pothole Region from 1974 - 2023 for adult female (**A**), juvenile female (**B**), adult male (**C**), and juvenile male (**D**) mallards from models not incorporating spatial variation.

**Figure 4.3.** Mean survival across the Prairie Pothole Region from 1974 - 2023 for adult female (**A**), juvenile female (**B**), adult male (**C**), and juvenile male (**D**) mallards from models not incorporating spatial variation.

**Figure 4.4.** Violin plots representing the posterior distribution of regression coefficients associated with natural mortality hazard rates for adult female (dark orange), adult male (dark blue), juvenile female (light orange), and juvenile male (light blue) mallards in the Prairie Pothole Region from 1974 - 2023. These regression coefficients come from the model not incorporating spatial variation.
